# ﻿*Lysimachiacavicola* (Primulaceae), a new species from Guangxi, China

**DOI:** 10.3897/phytokeys.235.109528

**Published:** 2023-11-17

**Authors:** You Nong, Yuan Fang, Chuan-Gui Xu, Gui-Yuan Wei, Ke-Jian Yan, Ren-Chuan Hu, Yuan-Guang Wen

**Affiliations:** 1 Guangxi Key Laboratory of Traditional Chinese Medicine Quality Standards, Guangxi Institute of Chinese Medicine & Pharmaceutical Science, No. 20-1 Dongge Road, Nanning, Guangxi, China Guangxi Key Laboratory of Traditional Chinese Medicine Quality Standards, Guangxi Institute of Chinese Medicine & Pharmaceutical Science Nanning China; 2 Guangxi Normal University, No.1 Yanzhong Road, Guilin, 541006, Guangxi, China Guangxi Normal University Guilin China; 3 Guangxi Academy of Sciences, No. 58 Daling Road, Nanning, 530007, Guangxi, China Guangxi Academy of Sciences Nanning China

**Keywords:** Conservation, dòng shēng xiāng cǎo, Fengshan, limestone, new species, taxonomy

## Abstract

*Lysimachiacavicola* (Subgen. Idiophyton, Primulaceae), a new species from Guangxi, China, is here described and illustrated based on morphological data. Although it shares similarities with *L.microcarpa*, *L.fooningensis*, and *L.capillipes*, there are distinguishing characteristics that set it apart. These include erect stems either solitary or in clusters of 1 to 2, herbaceous, terete, and densely glandular hairy. The leaves are either ovate or elliptical lanceolate, with inconspicuously reticulate veins. The petiole measures 2–4 mm in length covered with minute glandular hairy. The corolla is deeply parted, measuring 6–8 mm in length, with narrowly elliptic or narrowly oblong lobes that are 1–2 mm wide. The capsule is globose, measuring 2–3 × 2–3 mm, and possesses a chalky, brittle texture, which splits into 5-valved segments. The calyx of the plant appears yellowish-white during fruiting. This newly discovered species is endemic to limestone areas in Fengshan County, Guangxi, China.

## ﻿Introduction

The genus *Lysimachia* L. (1753: 146) was originally placed in Primulaceae (Cronquist 1981; Takhtajan 1997), but later transferred into Myrsinaceae, based on morphological and molecular evidence (Källersjö et al. 2000; Hao et al. 2004). Mysinaceae was later merged into Primulaceae s.l., hence *Lysimachia* was replaced into Primulaceae (Chen et al. 2016). *Lysimachia* L. is the largest genus in the tribe Lysimachieae (Primulaceae), which consists of approximately 200 species worldwide (Hao et al. 2004; Julius et al. 2016). According to the flower and gland morphology, the genus is separated into five subgenera, viz. subgen. Idiophyton Hand.–Mazz., subgen. Lysimachia, subgen. Palladia (Moench) Hand.–Mazz., subgen. Heterostylandra (Hand.–Mazz.) F.H.Chen & C.M.Hu and subgen. Naumburgia (Moench) Klatt. (Chen et al. 1989). The majority of species within the genus are distributed in temperate and subtropical regions of the Northern Hemisphere, with some species in Africa, Australia and South America. In China, the genus has 138 species and is highly diversified in south-western China, especially in limestone areas (Hu and Kelso 1996). Subsequently, more than 20 new species of *Lysimachia* have been discovered in the last two decades (e.g., Yan et al. 2017; Wang et al. 2018; Huang et al. 2019; Liu et al. 2020; Mou et al. 2020; Yi 2020; Ju et al. 2021; Lu et al. 2021; Yan et al. 2023).

The south-western limestone karst area is one of China’s biodiversity hotspots. Multiple field works have been conducted in this area in recent years. During fieldwork in April 2021 to Fengshan County in Southwest of China, we discovered an unknown species glowed at the cave entrance. After several observations and consulting relevant literature (Hu and Kelso 1996), we confirm that the unusual plant is a species new to science of *Lysimachia* and is described below.

## ﻿Materials and methods

### ﻿Morphology

The new species were described based on field observations that were conducted between April and August and examination of herbarium specimens at IBK and GXMI. Other related *Lysimachia* species were examined based on online images from the Kew Herbarium Catalogue (http://apps.kew.org/herbcat/gotoHomePage.do) and JSTOR Global Plants (http://plants.jstor.org/) and KUN, PE, IBSC and HITBC. Morphological characteristics that differentiate it from all other species in the genus of *Lysimachia* were used. The following characteristics were observed: stems, leaves, pedicels, flowers, receptacles, petals, stamens, gynoecium, carpels, size of flowers, size and shape of petals, number of stamens, and the shape of gynoecium and fruit. We also observed living plants of the new species during the flowering and fruiting period (April to July).

Descriptions were written from herbarium specimens. Measurements were made with a tape-measure and calipers. The structure of the indumentum and its distribution was observed and described under a dissecting microscope at magnifications of more than 20×. Additional information on locality, habitat, ecology, plant form and fruits were collected in the field and taken from herbarium labels. Conservation threat assessment followed IUCN Categories and Criteria (IUCN 2022).

## ﻿Results and discussion

### ﻿Taxonomy

#### ﻿ Lysimachia cavicola

Taxon classificationPlantaeEricalesPrimulaceae

Y.Nong & Y.G.Wen sp. nov.

7831FA46-6ECC-5206-91B0-1462CB57347D

urn:lsid:ipni.org:names:77331112-1

Figs 1 , 2 , 3 , 4

##### Chinese name.

dòng shēng xiāng cǎo (洞生香草).

##### Diagnosis.

*Lysimachiacavicola* shares several similarities with *L.microcarpa* C.Y.Wu, *L.fooningensis* C.Y.Wu, and *L.capillipes* Hemsl., including leaves alternate, flowers solitary in leaf axils, corolla yellow, anthers longer than filaments with distinct basifixation, and typically opening by apical pores, along with capsules dehiscing by valves. However, the new species can be easily distinguished by the following characteristics: stems solitary or clustered in 1–2, herbaceous and densely glandular; leaves ovate or elliptical lanceolate with inconspicuously reticulate veins; petioles 2–4 mm long that are minutely glandular hairy; capsules globose, 2–3 mm in diam., about the same length as the calyx. A more detailed morphological differences among the four species is presented in Table 1.

**Table 1.** Main morphological differences amongst *Lysimachiacavicola*, *L.microcarpa*, *L.fooningensis*, *L.capillipes*.

Morphological traits	*L.cavicola*	*L.microcarpa*	*L.fooningensis*	*L.capillipes*
Height	4–15 cm	10–30 cm	20–50 cm	40–60 cm
Stems	solitary or 1–2 in clusters, erect, terete and densely glandular	multiple clusters, ascending to erect, terete or weakly angular, upper part minutely glandular	solitary or 2–3 in clusters, erect, woody at base, terete or weakly angular in upper part, densely glandular	2 to many, erect, angular or winged, branched from middle, glabrous
Leaf blade	ovate or elliptical lanceolate, 1.5–3(4)× 0.5–1.6 cm; veins inconspicuously reticulate	ovate to rhomboid-ovate or ovate-elliptic, 1.5–3(–6) × 0.7–3 cm; veins prominently reticulate	elliptic-lanceolate to narrowly lanceolate, 3–11 × 0.7–2.8 cm; veins prominently reticulate	ovate to lanceolate, 1.5–3 × 1–3 cm; veins 4 or 5 pairs; veins inconspicuously reticulate
Petiole	2–4 mm, minutely glandular	4–8 mm, sparsely glandular	5–15 mm, sparsely glandular	2–8 mm, glabrous
Corolla	yellow, 6–8 mm, deeply parted; lobes narrowly elliptic or narrowly oblong, 5–8 × 1–2 mm, apex obtuse	yellow, 7–10 mm, deeply parted; lobes narrowly oblong, 6–9 × 2.7–4 mm, apex obtuse	yellow, 9–11 mm, deeply parted; lobes linear, 8–10 × 2–3 mm, apex obtuse	yellow, 6–8 mm, deeply parted; lobes narrowly oblong to linear, 5–7 × 1.8–3 mm, apex subobtuse
Calyx	lobes ovate-lanceolate, apex acuminate, 2 mm, surface glabrous except glandular hairy abaxially, margins sparsely glandular hairy, calyx yellowish-white in fruiting	lobes ovate, apex acuminate, 3.5–4 mm, surface glabrous, margins sparsely glandular hairy, calyx green in fruiting	lobes triangular, apex acuminate,2.5 mm, surface glabrous, margins sparsely glandular hairy, calyx green in fruiting	lobes ovate to lanceolate, apex acuminate to subulate, 2–4 mm, surface glabrous, margins minutely glandular hairy, calyx green in fruiting
Capsule	globose, 2–3 mm in diam., ca. as long as calyx	globose, 3–4 mm in diam., ca. as long as calyx	subglobose, 4 mm in diam., longer than calyx	globose, 3–4 mm in diam., longer than calyx

**Figure 1.**
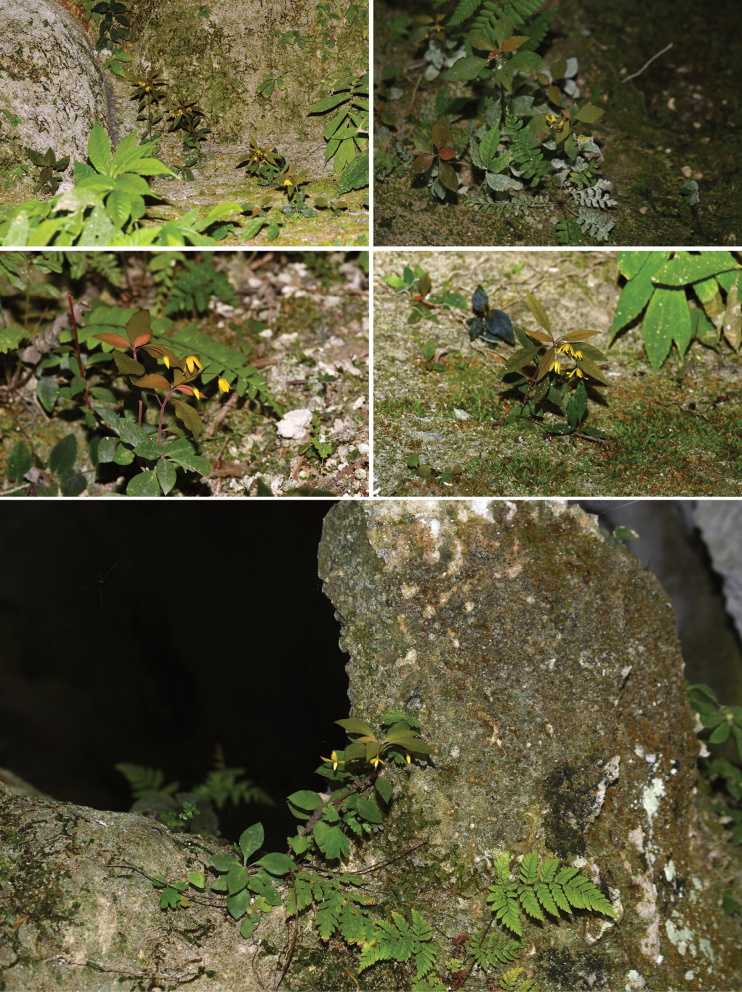
Habitat of *Lysimachiacavicola* at the entrance of a limestone cave. Photographed by YN and K–J Y.

##### Type.

China. Guangxi: Fengshan, limestone cave entrance, 24°34'17"N, 106°50'31"E, alt. 794 m, 23 April 2021 (fl.), *R.C. Hu HRC210423003* (holotype, GXMI!; isotypes, IBK!).

**Figure 2.**
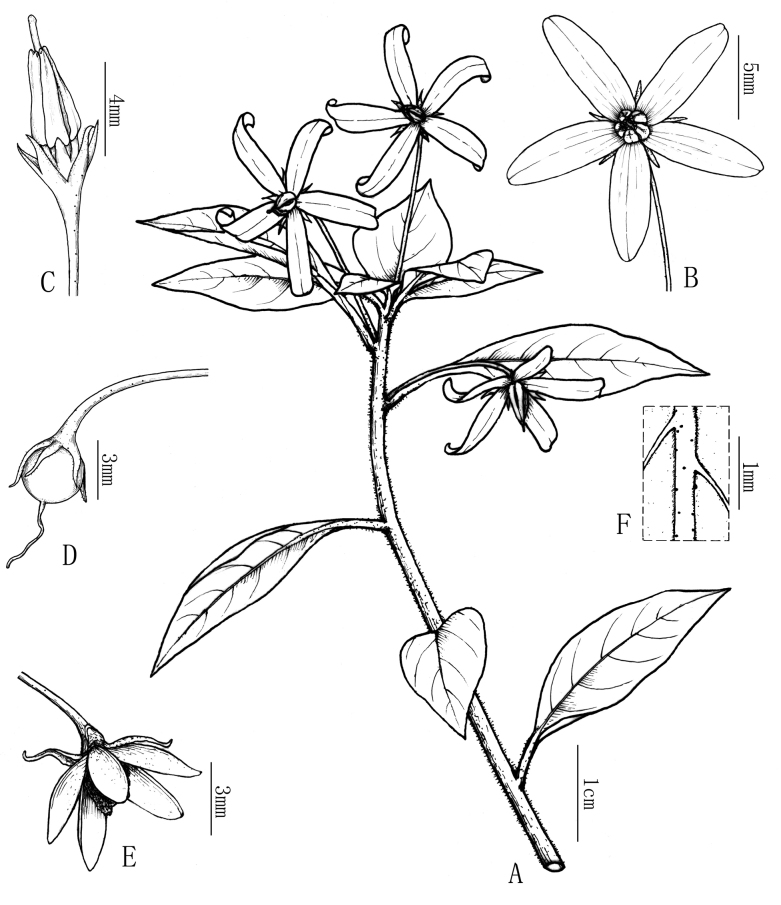
Line drawing of *Lysimachiacavicola***A** flowering branch **B** flower **C** anthers **D** fruit **E** capsule **F** mid vein abaxially. Drawn by X-CQ.

##### Description.

A perennial, herbaceous, 4–15 cm tall, aromatic when dry. ***Stems*** erect, solitary or 1–2 in clusters, terete and densely glandular. ***Leaves*** alternate: lamina ovate or elliptical lanceolate, 1.5–3(–4) × 0.5–1.6 cm, base attenuate, apex acuminate, glabrous except densely glandular hairy on midrid abaxially, veinlets invisible; petiole 2–4 mm long, minutely glandular. ***Flowers*** 5-merous, solitary, axillary at upper part of leaf axil; pedicel filiform, 2–4 cm long, sparsely glandular hairy; calyx green at flowering, lobes deeply parted, ovate-lanceolate, *c.* 2 mm long, glabrous except sparsely glandular abaxially and margin; corolla yellow, lobes deeply parted, narrowly elliptic or narrowly oblong, 6–8 × 1– 2 mm, apex recurved downward, glabrous on both surfaces; filaments short, not more than half the length of anthers, filaments connate basally into *c.* 1 mm high ring, free parts 1 mm; anthers 4 mm long, basifixed, opening by apical pores. ***Capsule*** globose, 2–3 × 2–3 mm, chalky, brittle, split into 5-valved, calyx yellowish-white in fruiting.

**Figure 3.**
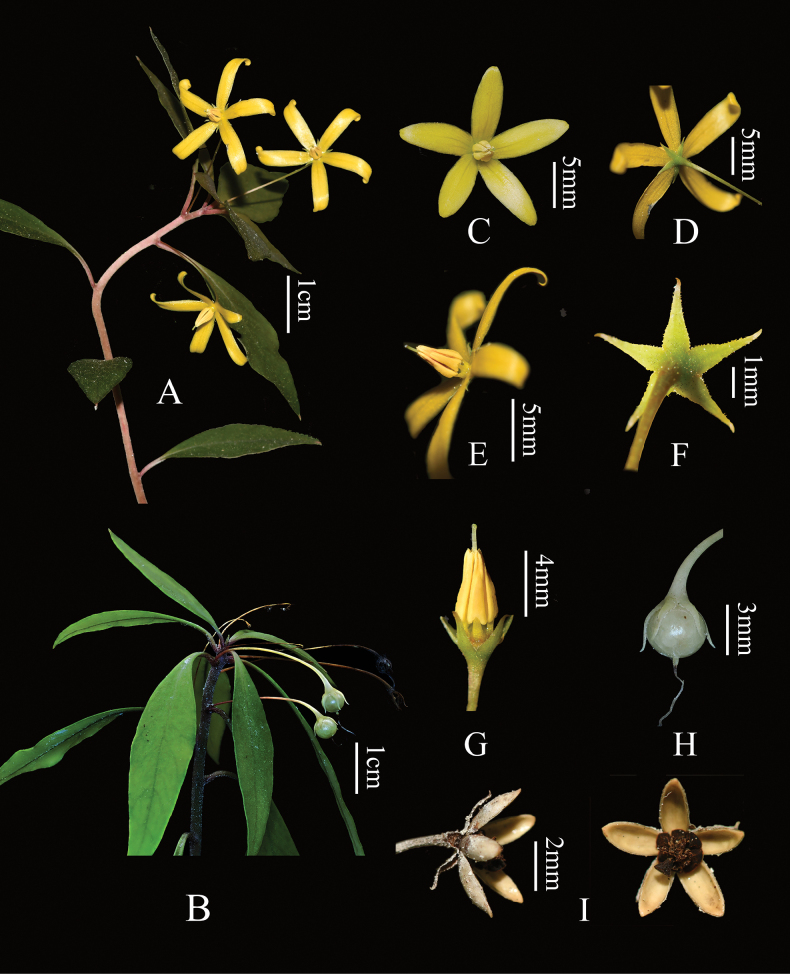
*Lysimachiacavicola***A** flowering plant **B** fruiting plant **C** flower (front view) **D** flower (back view) **E** flower (lateral view) **F** calyx (back view) **G** anthers **H** fruit **I** lateral (left) and front view (right) of capsule. Photographed by YN and K-JY.

##### Phenology.

Flowering in April–May; fruiting in June–July.

##### Etymology.

Fengshan, situated in the southwest of Guangxi, China, is a biodiversity hotspot known for its remarkable discoveries of new species (Nong et al. 2010; Wen et al. 2012; Li et al. 2019). One such species, *Lysimachiacavicola*, was found at the cave entrance in Fengshan, and the specific epithet is named after its habitat.

**Figure 4.**
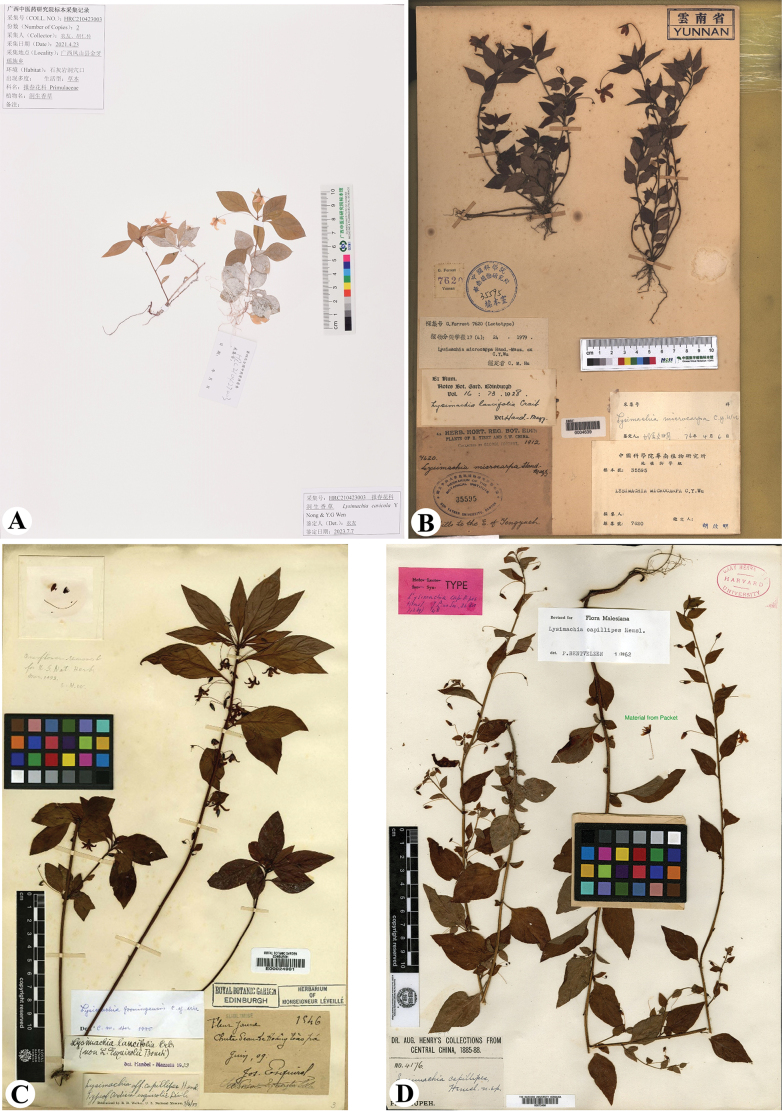
Digital images of type specimens **A***Lysimachiacavicola***B***L.microcarpa***C***L.fooningensis***D***L.capillipes*.

##### Distribution and habit.

**Figure 5.**
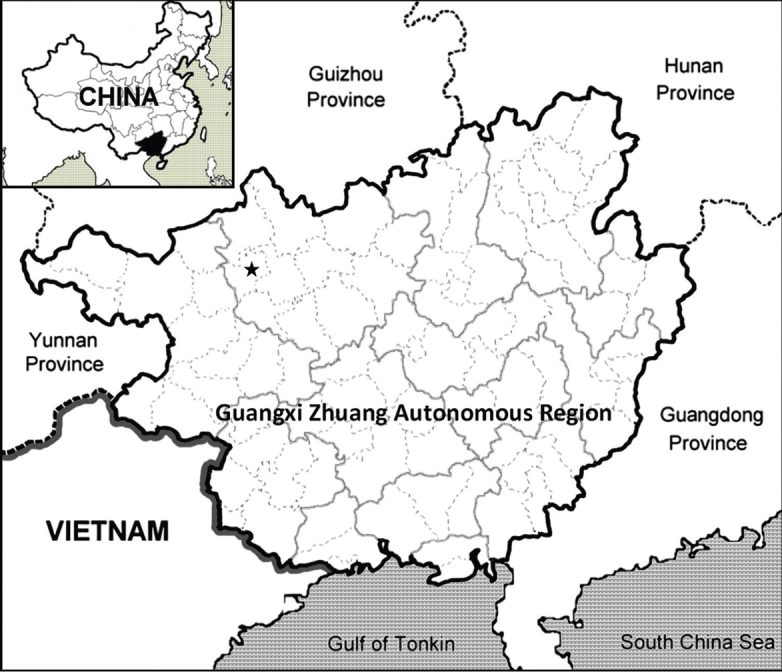
The distribution of *Lysimachiacavicola* (black pentagram) in Guangxi, China.

The new species is currently known only from the type locality from the southwest of Guangxi, China (Fig. 5). The new species mainly occurs at elevations of 800 m and it grows at the entrance of caves.

##### IUCN Red list category.

Data Deficient (DD). Data available for the new species are still insufficient to assess its conservation status. According to the IUCN criteria (IUCN 2022), it is considered Data Deficient until further information becomes available. Although *Lysimachiacavicola* currently has relatively good growth, further collecting and monitoring is necessary to allow more conclusive estimations about its rarity and vulnerability for future conservation planning of this species.

##### Additional specimen.

China. Guangxi: Fengshan, limestone cave entrance, 24°34'17"N, 106°50'31"E, alt. 794 m, 19 June 2023 (fr.), *You Nong NY20230619001* (GXMI!).

## ﻿Discussion

Based on the classification of *Lysimachia* by Handel-Mazzetti (1928) and Chen et al. (1989), the new species clearly belongs to LysimachiasubgenusIdiophyton sect. Apodanthera ser. Evalves Hand.–Mazz., which are characterised by corolla yellow, filaments connate into a thin ring and adnate at basal part of corolla tube, and the anthers longer than filaments, distinctly basifixed, opening by apical pores.

During our fieldwork, only one population with less than 50 individuals was discovered. All individuals were found growing at the cave entrance. More works such as conservation assessment and ex-situ conservation need to be done according to its limited populations, localities. Consequently, comprehensive surveys and studies on the phylogenetic evolution of *Lysimachia* within the limestone areas of southwest China will yield significant scientific insights into floristic geography and the phylogeny of *Lysimachia* in this particular region.

## Supplementary Material

XML Treatment for Lysimachia cavicola

## ﻿Additional information

### Author ORCIDs

You Nong https://orcid.org/0000-0001-7004-0946

Yuan Fang https://orcid.org/0009-0001-3637-0762

Chuan-Gui Xu https://orcid.org/0009-0000-6263-3821

Gui-Yuan Wei https://orcid.org/0000-0003-0652-1213

Ke-Jian Yan https://orcid.org/0000-0002-4927-4665

Ren-Chuan Hu https://orcid.org/0000-0002-0941-7203

Yuan-Guang Wen https://orcid.org/0000-0002-2493-1569

### Data availability

All of the data that support the findings of this study are available in the main text.
